# Antioxidant Metabolism and Chlorophyll Fluorescence during the Acclimatisation to Ex Vitro Conditions of Micropropagated *Stevia rebaudiana* Bertoni Plants

**DOI:** 10.3390/antiox8120615

**Published:** 2019-12-03

**Authors:** José Ramón Acosta-Motos, Laura Noguera-Vera, Gregorio Barba-Espín, Abel Piqueras, José A. Hernández

**Affiliations:** 1 Group of Fruit Tree Biotechnology, CEBAS-CSIC, 30100 Murcia, Spain; jacosta@cebas.csic.es (J.R.A.-M.); lauranoguera.ln@gmail.com (L.N.-V.); gbespin@cebas.csic.es (G.B.-E.); piqueras@cebas.csic.es (A.P.); 2 Cátedra Emprendimiento en el Ámbito Agroalimentario, Universidad Católica San Antonio de Murcia (UCAM) Campus de los Jerónimos, no. 135 Guadalupe, 30107 Murcia, Spain

**Keywords:** acclimatisation, antioxidant defences, chlorophyll fluorescence, in vitro culture, peroxidase, stevia plants

## Abstract

In this study, the functioning of antioxidant metabolism and photosynthesis efficiency during the acclimatisation of *Stevia rebaudiana* plants to ex vitro conditions was determined. A high percentage of acclimatised plants (93.3%) was obtained after four weeks. According to the extent of lipid peroxidation, an oxidative stress occurred during the first hours of acclimatisation. A lower activity of monodehydroascorbate reductase (MDHAR) than dehydroascorbate reductase (DHAR) was observed after 2 days of acclimatisation. However, after 7 days of acclimatisation, stevia plants activated the MDHAR route to recycle ascorbate, which is much more efficient energetically than the DHAR route. Superoxide dismutase and catalase activities showed a peak of activity after 7 days of acclimatisation, suggesting a protection against reactive oxygen species. Peroxidase activity increased about 2-fold after 2 days of acclimatisation and remained high until day 14, probably linked to the cell wall stiffening and the lignification processes. In addition, a progressive increase in the photochemical quenching parameters and the electronic transport rate was observed, coupled with a decrease in the non-photochemical quenching parameters, which indicate a progressive photosynthetic efficiency during this process. Taken together, antioxidant enzymes, lipid peroxidation, and chlorophyll fluorescence are proven as suitable tools for the physiological state evaluation of micropropagated plants during acclimatisation to ex vitro conditions.

## 1. Introduction

The application of in vitro culture techniques is a powerful vegetative proliferation tool for many plant species [1]. However, this process can be limited due to significant losses during acclimatisation to ex vitro conditions. For this raison, a better knowledge of the physiology and biochemistry of in vitro cultured plants that subsequently will be adapted to ex vitro conditions are of major interest. Light availability, and therefore the process of photosynthesis, is a key factor for ex vitro acclimatisation. Improvement of the photosynthetic activity is a critical step to reach a high survival rate during acclimatisation of in vitro plantlets [2]. In other words, proper photosynthesis activation is the key point to change the way to acquire carbon from heterotrophic or mixotrophic (in vitro conditions) to autotrophic (ex vitro conditions) sources. In grapevine, net photosynthesis and biomass are dependent on the increase in light intensity [2]. However, a distinct response was found in chestnut under the same conditions, where symptoms of photoinhibition were found during the acclimatisation process [2]. The transition from in vitro to ex vitro conditions has not been studied extensively, and an appropriate research model is still missing. Some of them have used the chlorophyll fluorescence technique as a non-destructive indicator to follow the acclimatisation process [1,2,3].

Micropropagated plants are very susceptible to environmental challenges after transferring to ex vitro conditions. For example, ex vitro plants are normally subjected to higher photosynthetic photon flux density (PPFD) than plants grown under in vitro conditions. In addition, the relative humidity (RH) is also lower under ex vitro conditions, thus plants are prone to suffer desiccation. Both phenomena, which contribute to photoinhibition damage and water stress, can induce the overproduction of reactive oxygen species (ROS). However, plants are provided with an efficient antioxidant defence mechanism to defend against the harmful effects of ROS. These defences include the ascorbate-glutathione (ASC-GSH) cycle enzymes (ascorbate peroxidase (APX), monodehydroascorbate reductase (MDHAR), dehydroascorbate reductase (DHAR) and glutathione reductase (GR)) and ROS-scavenging enzymes (superoxide dismutases (SODs), peroxidases (POX) and catalase (CAT)). The knowledge about the behaviour of the antioxidant machinery during ex vitro acclimatisation is very scarce, and only a few researchers have studied the changes on the enzymatic and non-enzymatic antioxidants during this process [1,3,4,5].

Stevia (*Stevia rebaudiana* Bertoni) is a perennial shrub belonging to the Asteraceae family. The leaves of *S. rebaudiana* contain a high concentration of steviol glycosides, stevioside and rebaudioside A being the prevalent forms, and used as natural sweeteners as a substitute for saccharose [6]. However, stevia seeds have little viability and the plant requires specific humidity, light, and nutrient conditions. The accumulation of steviol glycosides within *S. rebaudiana* is very variable due to significant genetic variability. The total steviol glycoside content changed not only between plants of the same cultivar, but also among similar plants in the same developmental stage [7]. In addition, a high antioxidant capacity of *S. rebaudiana* leaf extracts, related to their function as ROS-scavengers, has been reported [7,8,9]. These positive roles have been primarily associated with the presence of phenolic compounds [7]. Moreover, health-related effects of stevioside against type-II diabetes, hypertension, metabolic syndrome, and atherosclerosis have been reported [7]. Therefore, the production of in vitro clonal plants with a similar stevioside profile can be of commercial interest.

Accordingly, this work has focused on the acclimatisation to ex vitro conditions of stevia clones, originated from the micropropagation of plants previously characterised as high accumulators of steviol glycosides [10]. During the process of acclimatisation, the evolution of different parameters, including antioxidant metabolism, lipid peroxidation as an oxidative stress parameter, and chlorophyll fluorescence, were monitored to determine the oxidative stress that stevia plants might be suffering during the aforementioned process.

## 2. Material and Methods

### 2.1. Plant Material and Experimental Design

The plants were obtained from micropropagated stevia shoot cultures [10] (solid Murashige and Skoog (MS) medium supplemented with 60 mg L^−1^ phloroglucinol, 30 mg L^−1^ sequestrene, 0.8 mg L^−1^ meta-topolin, 6 mg L^−1^ adenine sulphate, 0.040 mg L^−1^ indole butyric acid, 3% sucrose, and a pH of 5.8). For elongation and rooting, shoots with three internodes were transferred to 1/2 MS medium without growth regulators, containing 40 mg L^−1^ sequestrene, 80 mg L^−1^ phloroglucinol, 250 mg L^−1^ MES buffer, 0.7% Agar, and a pH of 5.8. Under these conditions, the shoots elongated and rooted in 6 weeks. All cultures were maintained at 25 ± 2 °C in a growth chamber with a 16 h photoperiod (80 µmol m^−2^ s^−1^ photosynthetically active radiation, PAR). When the plantlets reached ca. 8–9 cm shoot length, the acclimatisation stage was initiated. For this, the rooted shoots were washed with distilled water to remove the agar and grown in an acclimatisation chamber (UBBINK propagator, (Northampton, UK)), consisting of a plastic tray and a transparent plastic cover, containing 2 vents for the control of the RH* in a mixture of perlite and peat (1:2, *v:v*). The substrate was moistened with distilled water and a systemic fungicide–bactericide (Beltanol-L, Probelte, Murcia, Spain) at 0.1% (*v/v*), which was also applied to the plantlets. These plantlets were kept in a culture chamber with a 16 h photoperiod and 25 °C, firstly at 150 μmoles cm^−2^ s^−1^ PAR for a period of ten days, followed by 18 days at 350 μmoles cm^−2^ s^−1^ PAR to complete the acclimatisation to ex vitro conditions. During this period, the respirators were gradually open to decrease the humidity progressively. For the different analysis, samples were taken from in vitro plantlets, and from plants at 2, 7, 14, 21, and 28 days of acclimatisation.

### 2.2. Measurement of Chlorophyll Fluorescence

Chlorophyll fluorescence was measured with a chlorophyll fluorimeter (IMAGIM-PAM M-series, Heinz Walz, Effeltrich, Germany) during the acclimatisation period of stevia plants to ex vitro conditions, at 2, 7, 14, 21, and 28 days of the initiation of the acclimatisation period. After a dark incubation period (20 min), the minimum and the maximal fluorescence yields of the stevia leaves were monitored. Kinetic analyses were carried out with actinic light (81 µmol quanta m^−2^ s^−1^ PAR) and repeated pulses of saturating light at 2700 µmol quanta m^−2^ s^−1^ PAR for 0.8 s, and at intervals of 20 s [11,12]. The following parameters were also analysed: effective PSII quantum yield (Y(II)); the quantum yield of regulated energy dissipation (Y(NPQ)); the non-photochemical quenching (NPQ); the maximal PSII quantum yield (Fv/ Fm); the coefficients of non-photochemical quenching (qN); the photochemical quenching (qP); and quantum yields of non-regulated energy dissipation Y(NO) [13].

### 2.3. Lipid Peroxidation

Stevia leaves were snap-frozen in liquid nitrogen and stored at −80 °C until use. The extent of lipid peroxidation was estimated by determining the concentration of thiobarbituric acid-reactive substances (TBARS) using a UV/Vis V-630 Bio spectrophotometer (Jasco, Tokyo, Japan). Leaf samples (0.2 g) were ground in liquid nitrogen into a fine powder and extracted in 1 M perchloric acid solution (1/10, w/v). Homogenates were centrifuged at 12,000× *g* for 10 min and 0.5 mL of the supernatant obtained was added to 1.5 mL 0.5% TBA in 1 M perchloric acid. The mixture was incubated at 90 °C in a shaking water bath for 20 min, and the reaction was stopped by placing the reaction tubes in an ice water bath. Then, the samples were centrifuged at 10,000× *g* for 5 min, and the absorbance of the supernatant was read at 532 nm. The value for non-specific absorption at 600 nm was subtracted. The amount of TBARS (red pigment) was calculated from the extinction coefficient 155 mM^−1^ cm^−1^ [10]. The lipid peroxidation was measured in plantlets under in vitro conditions as well as during the acclimatisation period of stevia plants to ex vitro conditions at 2, 7, 14, 21, and 28 days after the initiation of the acclimatisation process.

### 2.4. Enzyme Extraction and Analysis

Leaf samples were homogenized in liquid nitrogen and an extraction medium (1/5, *w/v*) containing 50 mM Tris-acetate buffer (pH 6.0); 0.1 mM EDTA; 2 mm cysteine; and 0.2 % (*v/v*) Triton X-100. For the ascorbate peroxidase (APX) activity, 20 mM sodium ascorbate was added to the extraction buffer. The extracts were centrifuged at 10,000× *g* for 20 min. The supernatant fraction was filtered on Sephadex NAP-10 columns (GE Healthcare, Chicago, IL, USA) equilibrated with the same buffer used for homogenisation and used for the enzymatic determinations. For the APX activity, 2 mM of sodium ascorbate was added to the equilibration buffer.

The enzymatic analyses were measured in plantlets under in vitro conditions as well as during the acclimatisation period of stevia plants to ex vitro conditions at 2, 7, 14, 21, and 28 days of the initiation of the acclimatisation process. The antioxidant enzyme determinations were carried according to protocols set up in our laboratory using a UV/Vis V-630 Bio spectrophotometer (Jasco) [11,14,15]. Specifically, APX (EC 1.11.1.11) was determined following the decrease at 290 nm due to the ascorbate oxidation by H_2_O_2_. MDHAR (EC 1.6.5.4) was determined following the decrease at 340 nm due to the NADH oxidation. DHAR (EC1.8.5.1) was determined by following the increase at 265 nm due to ascorbate formation. The reaction rate was corrected for the nonenzymatic reduction of DHA by reduced glutathione (GSH). GR (EC 1.6.4.2) was assayed by the decrease at 340 nm due to the NADPH oxidation. The reaction rate was corrected for the non-enzymatic oxidation of NADPH by oxidized glutathione (GSSH). SOD (EC 1.15.1.1) was assayed by the ferricytochrome c method using xanthine/xanthine oxidase as the source of superoxide radicals. CAT (EC 1.11.1.6) was measured following the decrease at 240 nm due to H_2_O_2_ consumption [14,15]. POX activity (EC. 1.11.1.7) was analysed following the oxidation of 4-methoxy-a-naphtol at 593 nm [15].

Protein contents were analysed according to [16] using a plate reader (Epoch2, BioTek, Winooski, VT, USA) and bovine serum albumin as standard.

### 2.5. Statistical Analysis

The data were analysed by one-way ANOVA followed by Tukey’s Multiple Range Test (*p* ≤ 0.05) to separate treatment means, using the SPSS 20.0 software (SPSS Inc., 2002, Chicago, IL, USA). Multivariate analysis using the StatGraphics Centurion XV software (StatPoint Technologies, Warrenton, VA, USA) were conducted by Principal Component Analysis (PCA), followed by a partial least squares discriminant analysis to assign the principal components displaying eigenvalues greater than or equal to 1.0.

## 3. Results

In the present work, the acclimatisation of in vitro *Stevia rebaudiana* Bertoni plants to ex vitro conditions was achieved with a high success rate, since 93.3% of the plants survived.

### 3.1. Chlorophyll Fluorescence Measurement

**Figure 1:**
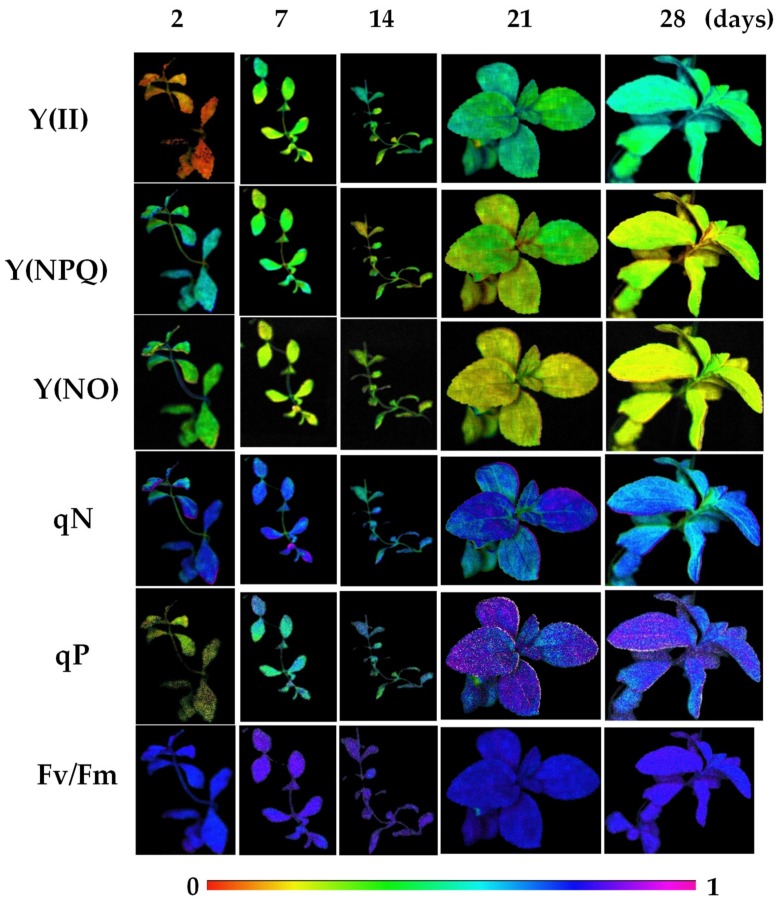
Evolution of the chlorophyll fluorescence parameters during the acclimatisation process of *S. rebaudiana* Bertoni plants to ex vitro conditions.

**Figure 2:**
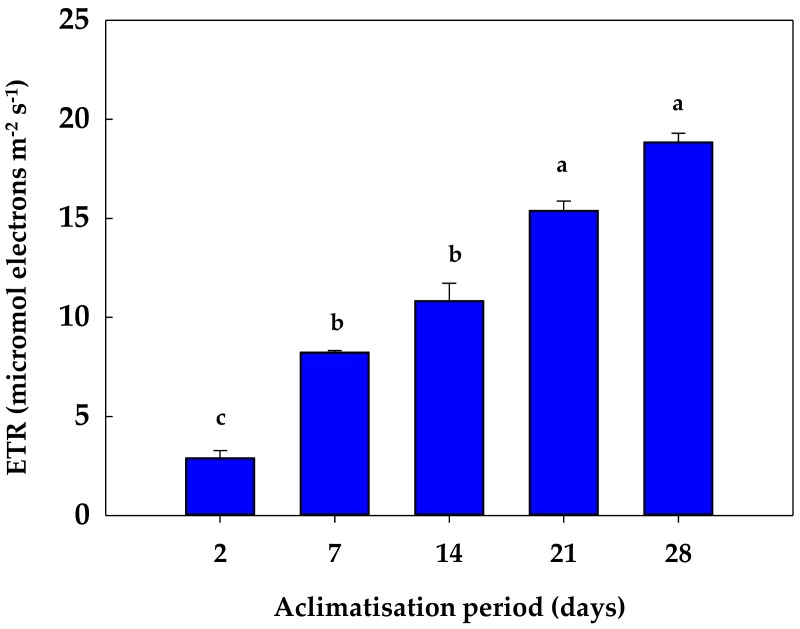
Evolution of the electron transport rate (ETR) during the acclimatization process of *S. rebaudiana* Bertoni plants to ex vitro conditions. Different letters in the same column indicate significant differences according to Tukey’s Multiple Range Test (*p* ≤ 0.05).

During the acclimatisation process, the evolution of the fluorescence parameters was monitored. At Day 2, the plants displayed higher values of the non-photochemical quenching parameters (Y(NPQ), Y(NO), NPQ and qN) and low values of the photochemical quenching parameters (Y(II), qP) (Table 1, Figure 1), as well as of the electron transport rate (ETR) (Figure 2). During the acclimatisation process, a progressive decrease in the non-photochemical quenching parameters and a constant increase in the photochemical-quenching parameters were observed. In that regard, Y(NPQ) continuously decreased, reducing its values by 40% and 50% after 21 and 28 days of the acclimatisation, respectively (Table 1, Figure 1). Concomitantly, Y(NO) declined during the acclimatisation assay, reaching a decrease near 40% and 30% after 21 and 28 days of acclimatisation, respectively (Table 1, Figure 1). NPQ displayed increases and decreases during the acclimatisation process. At first, after 7 days of acclimatisation this parameter increased by 22%. Then, the NPQ value increased by 63% after 14 days of acclimatization in relation to the precedent value (Day 7). One week later (day 21), again the NPQ value raised by 29% in comparison to the value observed in the second week (14 days). Finally, after 28 days of acclimatisation, a 30% decrease in the NPQ parameter was observed in relation to the value observed after 21 days (Table 1, Figure 1). However, although the qN values decreased during the acclimatisation process, the changes produced were not statistically significant (Table 1, Figure 1). Regarding the photochemical quenching parameters (Y(II) and qP), a progressive increase occurred during the acclimatisation. In both cases, the values increased near 3-fold after 7 and 14 days of acclimatisation, and about 5-fold after 21 and 28 days of the process (Table 1, Figure 1). Fv/Fm showed the lowest values after 2 days of acclimatisation. This parameter increased after 7 and 14 days, and then slightly decreased after 21 and 28 days of acclimatisation, but their values remaining statistically higher than the initial values (Table 1). The changes observed in Y(II) and qP correlated with the evolution of the ETR values, reaching an increase of near 6-fold at the end (28 days) of the acclimatisation process (Figure 2).

**Figure 3:**
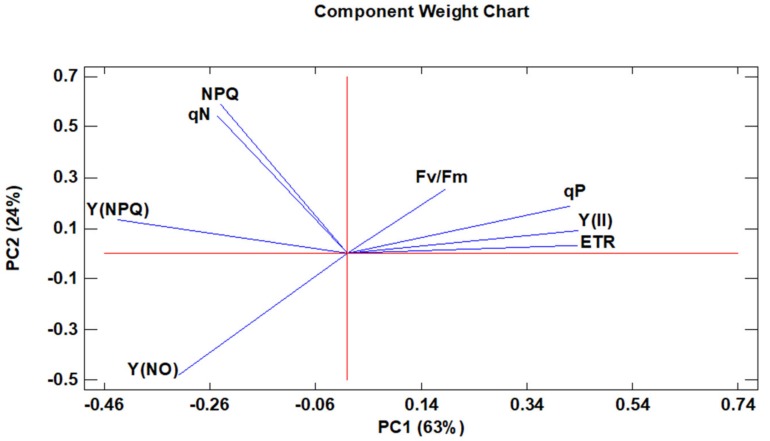
A principal component analysis applied to the fluorescence chlorophyll and electron transport rate (ETR) parameters. Two principal components (PC1 and PC2) resulted in a model that explained 87% of the total variance. The arrows denote eigen vectors characterised by the direction and the strength of the variable relative to PC1 and PC2.

Complementarily, PCA was utilized as a mathematical tool to determine associations among the different chlorophyll fluorescence parameters, ETR, and the acclimatisation evolution (Figure 3). The first component (PC1), which explains 63% of the variability of the experiment (Table S1, Table S2 and Figure S1), indicated the greater relevance of the photosynthetic efficiency mechanisms to adapt to ex vitro conditions, as reflected by the positive values that show all the measured parameters related to the photochemical quenching parameters, such as Y(II), qP and Fv/Fm, and ETR. On the contrary, the contribution of the non-photochemical quenching parameters, related to heat dissipation in a regulated or unregulated way, was negative in the PC1. This was the case for qN, the NPQ, and Y (NPQ) in the first case (regulated dissipation), and Y (NO) in the second case (unregulated dissipation). Regarding the second component (PC2), which explained 24% of the variability of the experiment (Table S1, Table S2 and Figure S1), it indicated that in a second level of importance for the acclimatisation to ex vitro conditions the excess of light energy was also relevant, being not destined to perform photosynthesis, but dissipated safely as heat by regulated mechanisms as observed in the high positive values that show all the related measured parameters (qN, NPQ and Y(NPQ)). Again, as in PC1, any unregulated heat dissipation mechanism played a harmful role in the plant as reflected in the negative values of Y(NO).

### 3.2. Antioxidant Metabolism

#### 3.2.1. Lipid Peroxidation Assay

**Figure 4:**
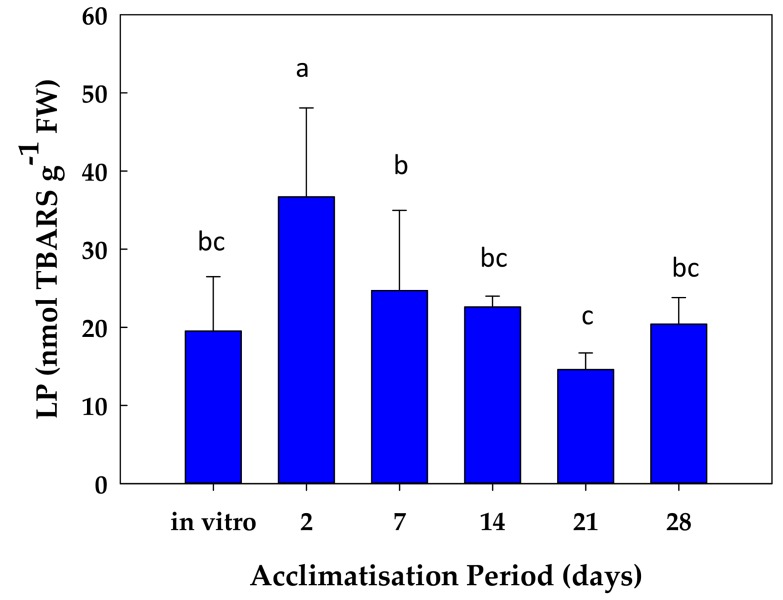
Levels of lipid peroxidation (LP) during the acclimatization process of *S. rebaudiana* Bertoni plants to ex vitro conditions. Different letters (a,b,c) in the same column indicate significant differences according to Tukey’s Multiple Range Test (*p* ≤ 0.05).

During the first hours of the acclimatisation process, stevia plants seemed to experience stress due to the modification on the culture conditions, as observed by the increase in the lipid peroxidation levels, measured as TBARS. In that regard, a peak after 2 days was detected, increasing by 86% with respect to values in the plantlet (Figure 4). Thereafter, and as the acclimatisation process to ex-vitro conditions progressed, the lipid peroxidation values progressively decreased reaching initial values (Figure 4).

#### 3.2.2. Antioxidant Enzymes

During the acclimatisation process of stevia plants to ex vitro conditions, the levels of some antioxidant enzymes, including the ASC-GSH cycle enzymes, superoxide dismutase (SOD), peroxidase (POX), and catalase (CAT), were analysed.

**Figure 5:**
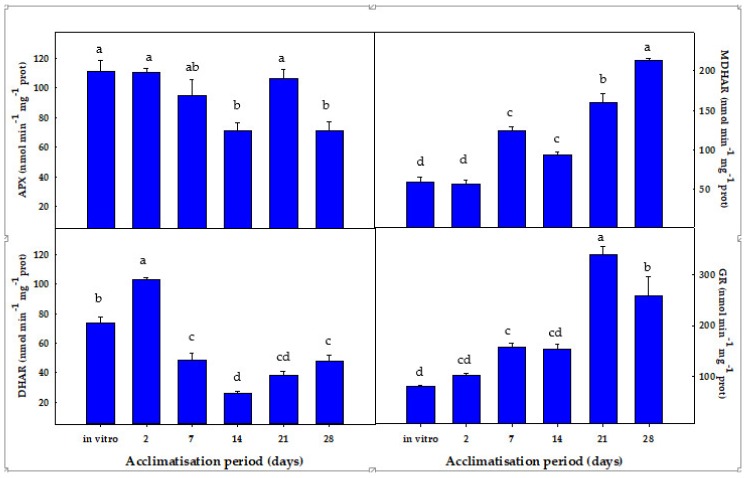
Evolution of the activity of the ASC-GSH cycle enzymes during the process of acclimatisation to ex vitro conditions of *S. rebaudiana* Bertoni plants. Different letters (a,b,c,d) in the same column indicate significant differences according to Tukey’s Multiple Range Test (*p* ≤ 0.05).

Regarding the ASC-GSH cycle enzymes, ascorbate peroxidase (APX) activity showed its highest activity in in vitro plants after 2 days of the acclimatisation process. The lowest APX activities were observed after 14 and 28 days, representing a decrease of ca. 35% with respect to the other time points (Figure 5). The monodehydroascorbate reductase (MDHAR) activity displayed its lower values at the beginning of the process of acclimatisation, i.e., under in vitro conditions and after 2 days of the acclimatisation process. Then, MDHAR activity progressively increased, especially after 21 and 28 days of acclimatisation, showing a 2.7- and 3.6-fold increase, respectively, with respect to values after 2 days (Figure 5). Regarding dehydroascorbate reductase (DHAR) activity, it behaved contrary to MDHAR activity. In that sense, DHAR showed its higher values after 2 days of the acclimatisation process, with a 40% increase in relation to the values observed in in vitro plants (Figure 5). Subsequently, DHAR activity progressively declined, with decreases ranging from 34% to 64% depending on the acclimatisation period, and a minimum DHAR activity observed after 14 days of acclimatisation (Figure 5). The GR activity behaved similarly to MDHAR activity. In that regard, GR increased as the plant acclimatisation process progressed, reaching their maximum values after 21 and 28 days of acclimatisation (4.2- and 3.2-fold increases, respectively) (Figure 5).

**Figure 6:**
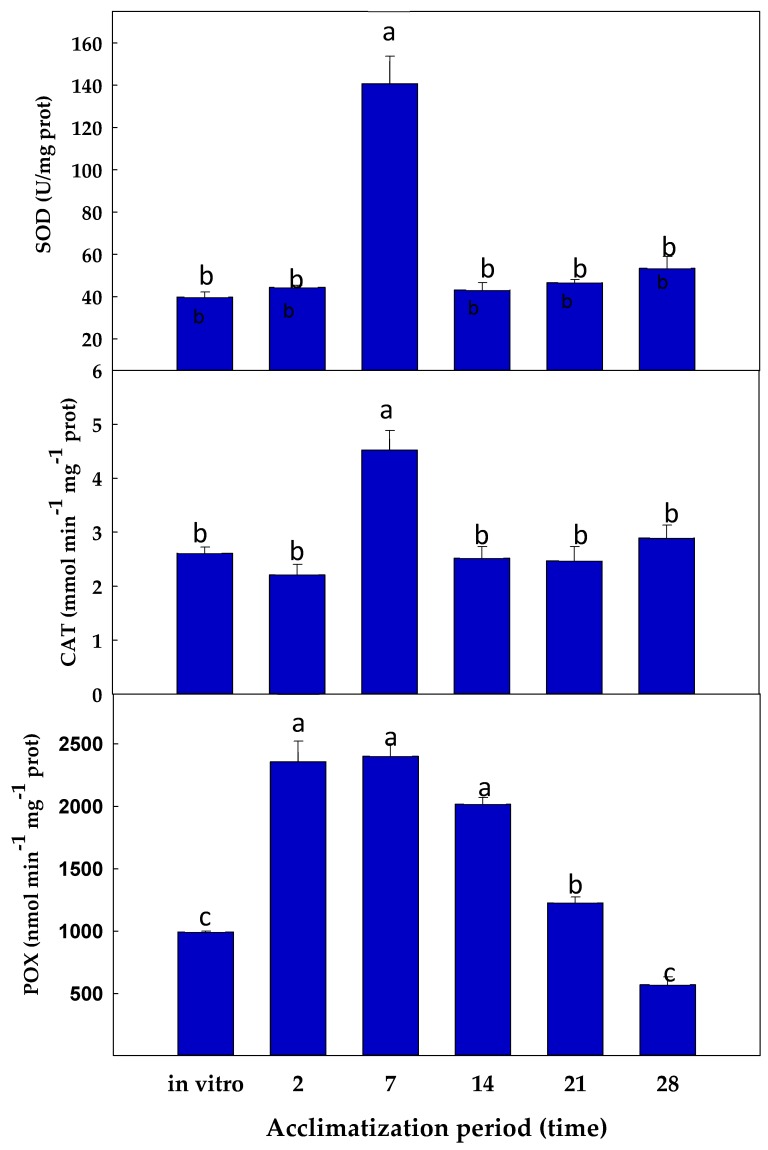
Evolution of the activity of SOD, CAT, and POX during the process of acclimatisation to ex vitro conditions of *S. rebaudiana* Bertoni plants. Different letters (a,b,c) in the same column indicate significant differences according to Tukey’s Multiple Range Test (*p* ≤ 0.05).

The activity SOD remained statistically invariable at all times except after 7 days of acclimatisation, where a 3.5-fold increase in relation to the initial values was observed (Figure 6). A similar response was observed for the CAT activity: a 1.7-fold increase occurred after 7 days of acclimatisation, whereas in the rest of the period the CAT activity displayed no significant differences in relation to the initial values (Figure 5). POX activity increased remarkably after 2 days, maintaining the value after 7 days (2.4-fold increase with respect to in vitro plants). Thereafter, POX activity progressively declined until reaching a 4.2-fold decrease with respect to the values after 7 days (Figure 6).

**Figure 7:**
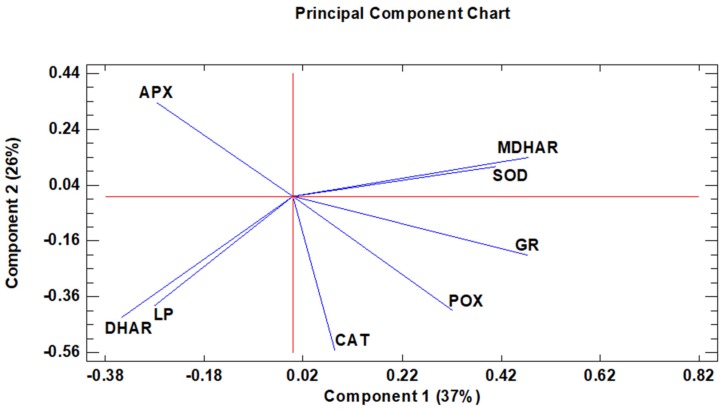
A principal component analysis applied to the antioxidant enzymes and lipid peroxidation variables. Two principal components (PC1 and PC2) resulted in a model that explained 63% of the total variance. The arrows denote eigen vectors characterised by the direction and the strength of the variable relative to PC1 and PC2.

Complementarily, a PCA study was carried out to analyse the associations between the different antioxidant enzymes monitored, as well as lipid peroxidation during the evolution of acclimatisation to ex vitro conditions (Figure 7). The resulting model with two components explained 63% of the total variance. The first component (PC1), which explained 37% of the variability within the dataset (Table S3, Table S4 and Figure S2), indicates the importance of the defence mechanisms to protect from the cellular damage associated with lipid peroxidation (LP), allowing a faster acclimatisation to ex vitro conditions. The efficient antioxidant mechanisms are reflected by the positive values observed in the ROS-eliminating enzymes (SOD, POX and CAT), as well as MDHAR and GR, which guarantees redox homeostasis of the plant. However, the contribution of DHAR and APX was less important, as denoted by their negative values. With respect to the second component (PC2), explaining 26% of the variability of the experiment (Tables S3 and S4), the loadings for LP, DHAR, CAT, and POX appeared as the dominant variables, and were opposite to the positive contribution of APX. Moreover, the loadings for DHAR and LP clustered together, which may relate to the parallel evolution of both variables during the acclimatisation period.

## 4. Discussion

The acclimatisation to ex vitro conditions is a critical step for the survival of micropropagated in vitro plants. Light availability is an important factor for a successful process of acclimatisation, involving a proper activation of the photosynthesis process [2]. The transition from in vitro to ex vitro conditions has been achieved in numerous plant species, both herbaceous and woody plants [2,4,8,10,17,18,19,20].

The micropropagation and acclimatisation of stevia plants can be an excellent tool to ensure the production of clonal, uniform, and true-to-type plant material. This may be used as a by-pass to the problems associated to the low germination rate and the great variation in the profile of steviol glycosides of *S. rebaudiana*. As a consequence, the production of uniform *S. rebaudiana* plants with the same steviol glycosides profile is of important commercial interest. This is particularly interesting for rebaudioside A, which provides a superior flavour to food products compared to other steviosides [6]. In a previous work, carried out in our laboratory, we reported that the main steviol glycoside detected in stevia plants after 10 week of acclimatisation was stevioside, whereas after 12 weeks, the main steviol glycoside was rebaudioside A, whose levels were not affected by the presence of NaCl [10] 

### 4.1. Chlorophyll Fluorescence

The use of the chlorophyll fluorescence technique has been used for different researchers to evaluate the acclimatisation process [1,2,3,21,22]. These authors found that acclimatised plants to ex vitro conditions increased the photosynthesis rate, which correlated with higher chlorophyll contents as well as Fv/Fm and Y(II) fluorescence parameters. In other studies, the authors used the photosynthesis rate or the chlorophyll a fluorescence technique, as well as the measurement of the antioxidant defences as indicators of the evolution of the acclimatisation process [1,2,3,4,17,19]. Accordingly, the monitoring of some physiological, biochemical, and molecular parameters can be useful tools to determine the success of ex vitro acclimatisation of in vitro plantlets.

Light intensity is another relevant abiotic factor influencing the acclimatization process. This was the case of grapevine and *Dieffenbachia* plantlets. In both cases, the increase in biomass or net photosynthesis was depending on the light intensity [2,5]. In grapevine, the photochemical quenching parameters, Y(II) and qP, showed higher values in the presence of 300 µmol m^−2^ s^−1^ of photons than in lower light intensities (100 µmol m^−2^ s^−1^), indicating a better photosynthetic efficiency at higher intensities. However, during the acclimatisation of *Dieffenbachia* plantlets, the Fv/Fm decreased with the increase in light intensity, demonstrating that photoinhibition occurred after transplanting micropropagated plants [5]. In grapevine, immediately after transferring to ex vitro conditions, a reversible photoinhibition occurred, as denoted by an initial decrease in Fv/Fm and F’q/F’m parameters followed by a progressive increased until Day 7 [3]. The mentioned responses could be due to the presence of poorly developed chloroplasts in the in vitro established leaves, resulting in a low resistance against photoinhibition [23].

In this work, a decrease in the non-photochemical quenching parameters and an increase in the photochemical-quenching parameters were observed during acclimatisation to ex vitro conditions. This response was correlated with a progressive increase in ETR values, indicating a gain of chloroplast efficiency during the process. In this sense, Y(II) represents the proportion of the light absorbed by chlorophyll associated with PSII that is used for photochemistry, whereas qP gives an indication of the proportion of the PSII reaction centres that are open [13].

In contrast, the high values observed in the non-photochemical quenching parameters in the first steps of the acclimatisation process would indicate that part of the light captured by the chloroplasts may dissipate as heat, protecting the chloroplast against the excess of radiation and avoiding the formation of ROS, especially singlet oxygen. Different authors have previously described the increase in non-photochemical parameters as an efficient mechanism for dissipating excess light energy and thus minimizing ROS generation [13]. In addition, it has been demonstrated that NPQ and Y(NPQ) are parameters very sensitive in the early detection of stress conditions by using fluorescence images [24]. Both NPQ and Y(NPQ) are related to the energy dissipated as heat by regulated mechanism (i.e., the xanthophyll cycle) [25]. In contrast, Y(NO) reflects the fraction of energy passively dissipated as heat and fluorescence, mainly due to closed PSII reaction centres. Therefore, high values of Y(NO) are related to the inability of plants to protects itself from excess light. In that regard, after 2 days of acclimatisation, stevia plants showed the highest values of Y(NO), which progressively decreased during the acclimatisation process, reflecting a better regulation [26]. On the other hand, high values of the non-photochemical parameters indicated that plants are suffering a stress. However, as the plant adapts to the new ex vitro conditions, these parameters decreased.

### 4.2. Antioxidant Response

Several authors used biochemical parameters, including antioxidant enzymes and lipid peroxidation, to follow the acclimation of in vitro plantlets. In the ornamental African violet, SOD, CAT, and glutathione-peroxidase (GSH-POX) increased after 28 days of acclimatisation, concomitantly with the increase in the light irradiance, suggesting that plants were suffering an abiotic stress [4]. However, the lipid peroxidation extent, measured as malondialdehyde contents, was 2-fold higher under low irradiance (35 µmol m^−2^ s^−1^) than in the presence of higher light intensities (70–100 µmol m^−2^ s^−1^), indicating the presence of an oxidative stress. This response was associated with a lower activity of the antioxidants enzymes as well as a low reduced glutathione (GSH) content. In fact, GSH not only acts as an antioxidant substrate in the ASC-GSH cycle but also as an antioxidant to minimize oxidative stress [27]. In a recent work, [5] analysed the activity of some antioxidant enzymes (SOD, CAT, and GSH-POX) during the acclimatisation of different *Dieffenbachia* cultivars grown at different light intensities. SOD and GSH-POX activity were much higher in the presence of the higher photosynthetic photon flux density (PPFD). However, the effect of PPFD on CAT activity was less evident, although the activity was higher in the presence of 100 PPFD (µmol m^−2^ s^−1^) than with lower light intensities (35 and 75 PPDF) [5]. According to these authors, this response of the antioxidant defences could be due to the higher ROS production induced by light stress.

In the present work, during the first days of ex vitro acclimatisation, an oxidative stress also occurred, as indicated by the lipid peroxidation levels. This variable is considered as an oxidative stress marker [12]; consequently, a damage to membrane lipids occurred, probably due to the change on the culture conditions. However, once this change on the culture conditions took place, the plants became progressively adapted to the new conditions, as reflected by the decrease in lipid peroxidation. A similar response has been described by [3] during the acclimatisation to ex vitro conditions of grapevine plantlets. These authors monitored the H_2_O_2_ levels, another oxidative stress marker, observing a 50% increase in H_2_O_2_ contents after 24 h of the acclimatisation process. Thereafter, H_2_O_2_ levels declined to the initial values and even lower [3]. In the same work, the increase in H_2_O_2_ after transfer to ex vitro conditions was followed by an increase in the expression of some antioxidant enzymes, including *APX1*, *GR1*, *SOD1*, and *SOD2* [3]. This response was partially similar to the observed in acclimatized stevia plants. In the present work, both MDHAR and GR activities increased during the acclimatisation process to ex vitro conditions, and other antioxidant enzymes as SOD, CAT, and POX peaked after 7 days of acclimatisation. In accordance with this, under in vitro conditions and after 2 days of acclimatisation, stevia plantlets displayed the highest DHAR activity, with values superior to MDHAR activity—the other ASC-recycling enzyme of the ASC-GSH cycle—at the same time. In that regard, after 2 days of acclimatisation the ratio DHAR/MDHAR was nearly 2. This suggests that, at that stage, DHAR activity is predominant in recycling ascorbate in stevia plants, using GSH as the electron donor. Subsequently, DHAR decreased and MDHAR progressively increased, reaching a DHAR/MDHAR ratio of 0.22 after 28 days of acclimatisation, where MDHAR activity was near 5-times higher than DHAR activity. Therefore, after 2 days of acclimatisation, stevia plants used the MDHAR way, spending NADH as a reducing power. It is necessary to clarify that the utilization of NADH to recycle the ASC is more efficient energetically than the use of GSH. Thus, different possibilities can be speculated to explain the higher DHAR activity under in vitro conditions and after 2 days of acclimatisation. The first is that under in vitro conditions, the culture media contained sucrose, so the plants had enough carbon source to generate energy through the glycolysis and respiration pathways, and thus they can afford the use GSH to recycle ASC (the inefficient way). The second possibility is that after 2 days of the acclimatisation process, the plants suffered an oxidative stress, as monitored by the lipid peroxidation data. Given that overexpression of *DHAR* has been associated with environmental stress tolerance [28], the higher DHAR activity observed at this stage could have a role coping with the stress resulting of the acclimatisation conditions. The third explanation is linked to the role of DHAR in plant growth and development [29]. Probably, after 2 days of acclimatisation the increased DHAR could have a function in plant growth and development processes. We also observed that MDHAR activity was enhanced after 7 days of acclimatisation. At this stage, photosynthesis seemed to work properly, as observed by the chlorophyll fluorescence and ETR values. Therefore, from that moment on, the plants produced their own sugars and energy to support plant growth. Probably, for this reason, plants changed the manner to recycle the ascorbate to an efficient way—via NADH.

On the other hand, it is known that DHAR and GR work together in the ASC-GSH cycle. We observed that under in vitro conditions and after 2 days of acclimatisation the ratio DHAR/GR was ca. 1, supporting the function of DHAR activity in the ascorbate recycling by using GSH as reducing power. However, from that moment on, the GR activity progressively increased, whereas DHAR significantly declined. This response supports the idea that most of the GSH produced in the GR reaction is used for other purposes, including the maintenance of redox homeostasis in stevia plants.

The peroxidase (POX) activity increased more than 2-fold after 2 days of acclimatisation and remained high until day 14, probably linked to the cell wall stiffening and the lignification processes that lead to hardening of the cell wall, as part of the plant differentiation process [30,31,32]. The overexpression of horseradish *POX* stimulated the growth of tobacco and hybrid aspen plants [33]. In pea seedlings and plants, a correlation between POX activity increase and growth was observed [34,35]. Thus, the observed increases in POX activity during the acclimatisation period can be linked to the plant growth and differentiation processes. However, a role of the POX eliminating H_2_O_2_ during the acclimatisation process cannot be ruled out. In fact, SOD, POX, and CAT displayed their maximum activities after 7 days of acclimatisation, indicating that these enzymes could work sequentially to eliminate O_2_•^−^ and H_2_O_2_ at that time, since SOD generates H_2_O_2_, which is eliminated by the action of the H_2_O_2_-scavenging enzymes, such as POX, CAT, or even APX, which maintained its activity after 7 days of acclimatisation. 

The results obtained by PCA confirmed the importance of the antioxidant mechanisms and the photosynthesis in the acclimatization of stevia plants. In general, greater efficiency in acclimatization to ex vitro conditions was more evident after 7 days of acclimatization and in the last two weeks (24 and 28 days), with increases in the antioxidant enzymes CAT, GR MDHAR, POX, and SOD, and also accompanied by decreases in lipid peroxidation (LP) in these same time periods. 

## 5. Conclusions

Taken together, the data suggested that antioxidant enzymes, lipid peroxidation, and chlorophyll fluorescence parameters can be suitable tools for the evaluation of the physiological state of micropropagated plants during the acclimatisation to ex vitro conditions of stevia plants, providing very useful information to monitor the stress state of the plants during the process of acclimatisation. This work has practical implications, since clonal plants of stevia with a known and stable profile of steviol glycosides are a suitable source of edulcorates and natural antioxidants to a diet.

## Figures and Tables

**Table 1:** Evolution of photochemical (Y(II), qP, Fv/Fm) Fv/Fm and non-photochemical quenching parameters (Y(NPQ), Y(NO), NPQ, qN) during the process of acclimatization to ex vitro conditions of *Stevia rebaudiana* Bertoni plants. ^a^ F: values from one-way ANOVA for the different chlorophyll fluorescence parameters at 99.9% level of significance (*).

Days	Y(II)	qP	Fv/Fm	Y(NPQ)	Y(NO)	NPQ	qN
**2**	0.090 ± 0.009c	0.160 ± 0.020c	0.747 ± 0.004c	0.544 ± 0.009a	0.393 ± 0.007a	0.354 ± 0.008b	0.719 ± 0.007a
**7**	0.256 ± 0.022b	0.445 ± 0.033b	0.782 ± 0.006ab	0.479 ± 0.020b	0.279 ± 0.008c	0.433 ± 0.017a	0.749 ± 0.012a
**14**	0.319 ± 0.048b	0.488 ± 0.038b	0.791 ± 0.005a	0.353 ± 0.023c	0.327 ± 0.012b	0.274 ± 0.019c	0.618 ± 0.022a
**21**	0.453 ± 0.014a	0.787 ± 0.013a	0.767 ± 0.002b	0.320 ± 0.016cd	0.226 ± 0.004d	0.355 ± 0.023b	0.706 ± 0.017a
**28**	0.446 ± 0.009a	0.710 ± 0.018a	0.772 ± 0.003b	0.272 ± 0.006d	0.282 ± 0.011c	0.248 ± 0.012c	0.605 ± 0.014a
**^a^ F**	70.82*	88.59*	14.89*	62.85*	40.08*	25.81*	21.73*

Different letters in the same column indicate significant differences according to Tukey’s Multiple Range Test (*p* ≤ 0.05).

## Supplementary Materials

The following are available online at https://www.mdpi.com/2076-3921/8/12/615/s1, Figure S1: Sedimentation graph where two PCA with eigenvalues greater than or equal to 1.0 were obtained to determine associations among the different chlorophyll fluorescence parameters, ETR and the acclimatisation evolution, Figure S2: Sedimentation graph where two PCA with eigenvalues greater than or equal to 1.0 were obtained to analyse the associations between the different antioxidant enzymes monitored, as well as lipid peroxidation during the evolution of acclimatisation to ex vitro conditions, Table S1: Weight of the Components for the fluorescence chlorophyll parameters and ETR, Table S2: Eigenvalues and percentage of variance of components for the fluorescence chlorophyll parameters and ETR, Table S3: Weight of the Components for the antioxidant enzymes and lipid peroxidation data (LP), Table S4: Eigenvalues and percentage of variance of components for the antioxidant enzymes and lipid peroxidation data.

## References

[1] Van-Huylenbroeck J.M., Piqueras A., Debergh P.C. (2000). The evolution of photosynthetic capacity and the antioxidant enzymatic system during acclimatization of micropropagated *Calathea* plants. Plant Sci..

[2] Carvalho L.C., Osorio M.L., Chaves M.M., Amâncio S. (2001). Chlorophyll fluorescence as an indicator of photosynthetic functioning of in vitro grapevine and chestnut plantlets under ex vitro acclimatization. Plant Cell Tissue Organ Cult..

[3] Carvalho L.C., Vilela B.J., Vidigal P., Mullineaux P.M., Amâncio S. (2006). Activation of the ascorbate-glutathione cycle is an early response of micropropagated *Vitis vinifera* L. explants transferred to ex Vitro. Int. J. Plant Sci..

[4] Dewir Y.H., El-Mahrouk M.E., Al-Shmgani H.S., Rihan H.Z., Teixeira da Silva J.A., Fuller M.P. (2015). Photosynthetic and biochemical characterization of in vitro-derived African violet (*Saintpaulia ionantha* H. Wendl) plants to ex vitro conditions. J. Plant Interact..

[5] El-Mahrouk M.E., Dewir Y.H., Murthy H.N., Rihan H.Z., Al-Shmgani H.S., Fuller M.P. (2016). Effect of photosynthetic photon flux density on growth, photosynthetic competence and antioxidant enzymes activity during ex vitro acclimatization of *Dieffenbachia* cultivars. Plant Growth Regul..

[6] Zeng J., Cheng A., Lim D., Yi B., Wu W. (2013). Effects of salt stress on the growth, physiological responses, and glycoside contents of *Stevia rebaudiana* Bertoni. J. Agric. Food Chem..

[7] Ceunen S., Geuns J.M.C. (2013). Steviol Glycosides: Chemical Diversity, Metabolism, and Function. J. Nat. Prod..

[8] Ghanta S., Banerjee A., Poddar A., Chattopadhyay S. (2007). Oxidative DNA Damage Preventive Activity and Antioxidant Potential of *Stevia rebaudiana* (Bertoni) Bertoni, a Natural Sweetener. J. Agric. Food Chem..

[9] Jahan I.A., Mostafa M., Hossain H., Nimmi I., Sattar A., Alim A., Moeiz S.M.I. (2010). Antioxidant activity of *Stevia rebaudiana* Bert. leaves from Bangladesh. Bangladesh Pharm. J..

[10] Cantabella D., Piqueras A., Acosta-Motos J.R., Bernal-Vicente A., Hernandez J.A., Diaz-Vivancos P. (2017). Salt-tolerance mechanisms induced in *Stevia rebaudiana* Bertoni: Effects on mineral nutrition, antioxidative metabolism and steviol glycoside content. Plant Physiol. Biochem..

[11] Acosta-Motos J.R., Díaz-Vivancos P., Álvarez S., Fernández-García N., Sánchez-Blanco M.J., Hernández J.A. (2015). Physiological and biochemical mechanisms of the ornamental *Eugenia myrtifolia* L. plants for coping with NaCl stress and recovery. Planta.

[12] Acosta-Motos J.R., Díaz-Vivancos P., Álvarez S., Fernández-García N., Sánchez-Blanco M.J., Hernández J.A. (2015). NaCl-induced physiological and biochemical adaptative mechanisms in the ornamental *Myrtus communis* L. plants. J. Plant Physiol..

[13] Maxwell K., Johnson G.N. (2000). Chlorophyll fluorescence: A practical guide. J. Exp. Bot..

[14] Ros Barceló A., Gómez-Ros L.V., Ferrer M.A., Hernández J.A. (2006). The apoplastic antioxidant enzymatic system in the wood-forming tissues of trees. Trees.

[15] Barba-Espín G., Clemente-Moreno M.J., Álvarez S., García-Legaz M.F., Hernández J.A., Díaz-Vivancos P. (2011). Salicylic acid negatively affects the response to salt stress in pea plants: Effects on *PR1b* and *MAPK* expression. Plant Biol..

[16] Bradford M.M. (1976). A rapid and sensitive method for the quantitation of microgram quantities of protein utilizing the principle of protein-dye binding. Anal. Biochem..

[17] Ďurkovič J., Čaňová I., Pichler V. (2009). Water loss and chlorophyll fluorescence during ex vitro acclimatization in micropropagated black mulberry (*Morus nigra* L.). Propag. Ornam. Plants.

[18] Clemente-Moreno M.J., Piqueras A., Hernández J.A. (2011). Implication of peroxidase activity in development of healthy and PPV-infected micropropagated GF305 peach plants. Plant Growth Regul..

[19] Chaari-Rkhis A., Maalej M., Chelli-Chaabouni A., Fki L., Drira N. (2015). Photosynthesis parameters during acclimatization of in vitro-grown olive plantlets. Photosynthetica.

[20] Diaz-Vivancos P., Faize L., Nicolas E., Clemente-Moreno M.J., Bru-Martinez R., Burgos L., Hernández J.A. (2016). Transformation of plum plants with a cytosolic ascorbate peroxidase transgene leads to enhanced water stress tolerance. Ann. Bot..

[21] Pospisilova J., Ticha I., Kadlecek P., Haisel D., Plazakova S. (1999). Acclimatization of micropropagated plants to ex vitro conditions. Biol. Plant..

[22] Van-Huylenbroeck J.M., Piqueras A., Debergh P.C. (1998). Photosynthesis and carbon metabolism in leaves formed prior and during ex vitro acclimatization of micropropagated plants. Plant Sci..

[23] Lee N., Wetzstein H.Y., Sommer H.E. (1985). Effects of quantum flux density on photosynthesis and chloroplast ultrastrucutre in tissue-cultured plantlets and seedling of *Liquidambar styraciflua* L. towards improved acclimatization and filed survival. Plant Physiol..

[24] Pérez-Bueno M.L., Ciscato M., vandeVen M., García-Luque I., Barón M., Valcke R. (2006). Imaging viral infection: Studies on *Nicotiana benthamiana* plants infected with the pepper mild mottle virus. Photosynth. Res..

[25] Zhang Q.Y., Wang L.Y., Kong F.Y., Deng Y.S., Li B., Meng Q.W. (2012). Constitutive accumulation of zeaxanthin in tomato alleviates salt stress-induced photoinhibition and photooxidation. Physiol. Plant..

[26] Klughammer C., Schreiber U. (2008). Complementary PSII quantum yields calculated from simple fluorescence parameters measured by PAM fluorometry and the saturation pulse method. PAM Appl. Notes (PAN).

[27] Alscher R.G., Donahue J.L., Cramer C.L. (1997). Reactive oxygen species and antioxidant: Relationship in green cells. Physiol. Plant..

[28] Eltayeb A.E., Kawano N., Badawi G.H., Kaminaka H., Sanekata T., Morishima I., Shibahara T., Inanaga S., Tanaka K. (2006). Enhanced tolerance to ozone and drought stresses in transgenic tobacco overexpressing dehydroascorbate reductase in cytosol. Physiol. Plant..

[29] Potters G., Horemans N., Caubergs R.J., Asard H. (2012). Ascorbate and dehydroascorbate influence cell cycle progression in a tobacco cell suspension. Plant Physiol..

[30] Pomar F., Caballero N., Pedreño M.A., Ros Barceló A. (2002). H_2_O_2_ generation during the auto-oxidation of coniferyl alcohol drives the oxidase activity of a highly conserved class III peroxidase involved in lignin biosynthesis. FEBS Lett..

[31] Sato Y., Demura T., Yamawaki K., Inoue Y., Sato S., Sugiyama M., Fukuda H. (2006). Isolation and characterization of a novel peroxidase gene ZPO-C whose expression and function are closely associated with lignification during tracheary element differentiation. Plant Cell Physiol..

[32] Boerjan W., Ralph J., Baucher M. (2003). Lignin biosynthesis. Annu. Rev. Plant Biol..

[33] Kawaoka A., Matsunaga E., Endo S., Kondo S., Yoshida K., Shinmyo A., Ebinuma H. (2003). Ectopic expression of a horseradish peroxidase enhances growth rate and increases oxidative stress resistance in hybrid aspen. Plant Physiol..

[34] Dıaz-Vivancos P., Barba-Espín G., Clemente-Moreno M.J., Hernandez J.A. (2010). Characterization of the antioxidant system during the vegetative development of pea plants. Biol. Plant..

[35] Barba-Espín G., Diaz-Vivancos P., Clemente-Moreno M.J., Albacete A., Faize L., Faize M., Perez-Alfocea F., Hernandez J.A. (2010). Interaction between hydrogen peroxide and plant hormones during germination and the early growth of pea seedlings. Plant Cell Environ..

