# Supplementary material for: Antioxidant Metabolism and Chlorophyll Fluorescence during the Acclimatisation to Ex Vitro Conditions of Micropropagated Stevia rebaudiana Bertoni Plants
Source: Antioxidants (Basel). 2019 Dec 3;8(12):615. doi: 10.3390/antiox8120615 (PMC6943525; doi:10.3390/antiox8120615)
Supplement: Supplementary file 1 [file antioxidants-08-00615-s001.pdf]

Table S1. Weight of the Components for the fluorescence chlorophyll parameters and ETR.

Parameters	Component 1	Component 2
Y(II)	0.437	0.089
Y(NPQ)	-0.434	0.132
Y(NO)	-0.318	-0.479
NPQ	-0.239	0.590
qN	-0.246	0.544
qP	0.422	0.188
ETR	0.436	0.032
Fv/Fm	0.185	0.254

Table S2. Eigenvalues and percentage of variance of components for the fluorescence chlorophyll parameters and ETR.

Component	Eigenvalue	Percentage of Variance	Percentage Accumulated
1	5.013	62.662	62.662
2	1.901	23.761	86.423
3	0.789	9.861	96.284
4	0.191	2.386	98.670
5	0.053	0.667	99.337
6	0.032	0.399	99.737
7	0.012	0.147	99.884
8	0.009	0.116	100.000

Table S3. Weight of the Components for the antioxidant enzymes and lipid peroxidation data (LP).

Variable	Component 1	Component 2
APX	-0.276	0.336
POX	0.320	-0.408
CAT	0.084	-0.552

DHAR	-0.347	-0.433
MDHAR	0.474	0.139
GR	0.473	-0.212
SOD	0.409	0.107
LP	-0.280	-0.391

Table S4. Eigenvalues and percentage of variance of components for the antioxidant enzymes and lipid peroxidation data.

Component	Eigenvalue	Percentage of Variance	Percentage Accumulated
1	2.990	37.378	37.378
2	2.063	25.792	63.170
3	0.856	10.697	73.867
4	0.714	8.923	82.789
5	0.570	7.125	89.914
6	0.362	4.529	94.443
7	0.298	3.726	98.170
8	0.146	1.830	100.000

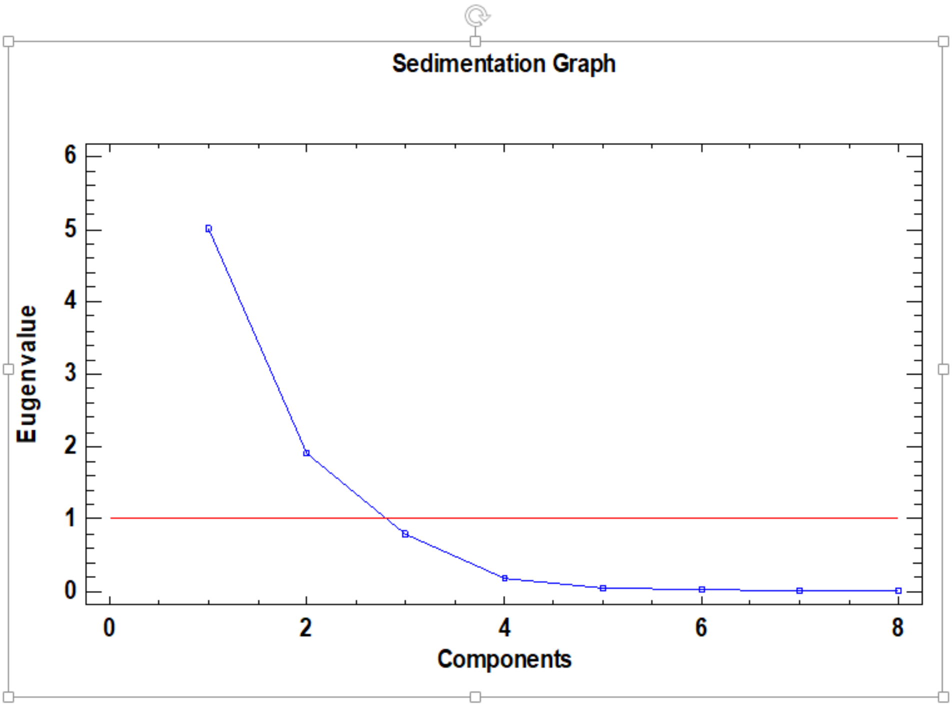

Figure 1. Sedimentation graph where two PCA with eigenvalues greater than or equal to 1.0 were obtained to determine associations among the different chlorophyll fluorescence parameters, ETR and the acclimatisation evolution.

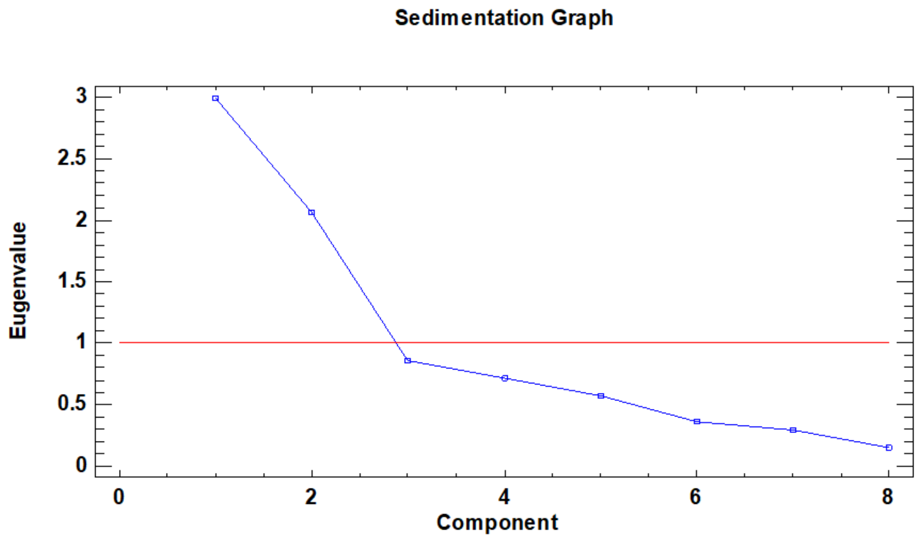

Figure S2. Sedimentation graph where two PCA with eigenvalues greater than or equal to 1.0 were obtained to analyse the associations between the different antioxidant enzymes monitored, as well as lipid peroxidation during the evolution of acclimatisation to *ex vitro* conditions
